# Systematic Review of Mental Health Challenges Among Female Sex Workers: A Focus on Risks and Barriers

**DOI:** 10.1192/j.eurpsy.2026.11941

**Published:** 2026-07-31

**Authors:** E. V. Neira, C. L. González

**Affiliations:** Hospital Clínico Universitario de Santiago de Compostela, Santiago de Compostela, Spain

## Abstract

**Introduction:**

Female sex workers (FSWs) encounter a range of occupational hazards including violence, high-risk sexual practices, exploitation, and a lack of legal safeguards. These challenges contribute significantly to their mental health burden, yet this area remains under-researched. This review aims to consolidate knowledge on the mental health of FSWs and identify key risk factors and barriers to accessing care.

**Objectives:**

The main objective is to assess the prevalence and types of mental health issues among FSWs. Secondary goals include exploring personal and occupational risk factors, the role of societal stigma, and barriers to accessing mental health services.

**Methods:**

A systematic review was conducted, registered with PROSPERO, and involved a comprehensive search of Web of Science, PubMed, Scopus, and PsycInfo for studies from 2010 to 2022. Inclusion criteria focused on quantitative studies concerning FSWs over 18, with publications in English or Spanish. Thirty studies were analyzed, covering a total sample of 19,507 participants, primarily female.

**Results:**

Prevalent mental health disorders included depression (50%-88%), anxiety (13.6%-51%), and PTSD (10%-39.6%), often linked to childhood sexual abuse, which was reported by over 50% of FSWs in some studies. Substance use disorders were also notable, with problematic alcohol use ranging from 36.7% to 45.4%, and illicit drug use from 20.9% to 32.7%. Risk factors such as being female, young, part of an ethnic minority, or lacking social support were significant. Occupational risks included frequent experiences of violence, especially among transgender FSWs, and poor working conditions, which exacerbated mental health issues. Barriers to accessing mental health care included institutional stigma, discrimination, and legal challenges.

**Conclusions:**

FSWs experience disproportionately high rates of mental health issues compared to the general population, driven by a combination of personal, occupational, and societal factors. Addressing these challenges requires targeted interventions that reduce stigma, improve access to mental health care, and provide legal protection. There is a critical need for more longitudinal and gender-inclusive research to inform effective policies and interventions tailored to the unique needs of FSWs.

**Disclosure of Interest:**

None Declared

